# Computational Hardness of Collective Coin-Tossing Protocols

**DOI:** 10.3390/e23010044

**Published:** 2020-12-30

**Authors:** Hemanta K. Maji

**Affiliations:** Department of Computer Science, Purdue University, West Lafayette, IN 47907, USA; hmaji@purdue.edu; Tel.: +1-765-494-6184

**Keywords:** collective coin-tossing, full information model, optimal coin-tossing protocols, isoperimetric inequalities, relativized separation, black-box separation

## Abstract

Ben-Or and Linial, in a seminal work, introduced the full information model to study collective coin-tossing protocols. Collective coin-tossing is an elegant functionality providing uncluttered access to the primary bottlenecks to achieve security in a specific adversarial model. Additionally, the research outcomes for this versatile functionality has direct consequences on diverse topics in mathematics and computer science. This survey summarizes the current state-of-the-art of coin-tossing protocols in the full information model and recent advances in this field. In particular, it elaborates on a new proof technique that identifies the minimum insecurity incurred by any coin-tossing protocol and, simultaneously, constructs the coin-tossing protocol achieving that insecurity bound. The combinatorial perspective into this new proof-technique yields new coin-tossing protocols that are more secure than well-known existing coin-tossing protocols, leading to new isoperimetric inequalities over product spaces. Furthermore, this proof-technique’s algebraic reimagination resolves several long-standing fundamental hardness-of-computation problems in cryptography. This survey presents one representative application of each of these two perspectives.

## 1. Introduction

Ben-Or and Linial [1,2], in a seminal work, introduced the full information model to study collective coin-tossing protocols. Collective coin-tossing protocols upgrade the local independent private randomness of each of the processors into shared randomness with which all processors agree. In this model, each processor has an unbounded computational power and communicates over a broadcast channel. Collective coin-tossing is an elegant functionality providing uncluttered access to the primary bottlenecks of achieving security in a specific adversarial model. These hardness of computation results for the coin-tossing functionality extend to other general functionalities as well. Furthermore, the research outcomes for this functionality has direct consequences on diverse topics in mathematics and computer science—for example, extremal graph theory [3,4,5], extracting randomness from imperfect sources [6,7,8,9], cryptography [10,11,12,13,14,15,16], game theory [17,18], circuit representation [19,20,21], distributed protocols [22,23,24], and poisoning and evasion attacks on learning algorithms [25,26,27,28].

This survey summarizes the current state-of-the-art of coin-tossing protocols in the full information model and recent advances in this field, settling several long-standing open problems. In particular, it elaborates on a new proof technique introduced in [14] to simultaneously characterize the optimal coin-tossing protocols and prove lower bounds to the insecurity of any coin-tossing protocol. The geometric (or combinatorial) interpretation of this proof technique is inherently constructive; that is, the proof technique identifies the optimal coin-tossing protocols, which have applications in new isoperimetric inequalities in the product spaces over large alphabets [29]. This proof technique’s algebraic reimagination lifts these hardness of computation results to more complex relativized settings via a new data processing inequality, central to resolving some of the most fundamental problems in computer science and cryptography [15,16,30].

1. Geometric/combinatorial proof-technique. Section 3 models a coin-tossing protocol as a martingale that evolves from $X_{0}\in \left(0,1\right)$ to $X_{n}\in \left\{0,1\right\}$ in n∈ℕ discrete time-steps. Any stopping time τ∈{1,2,…,n,∞} in this coin-tossing martingale translates into adversarial attacks on the coin-tossing protocol. Khorasgani, Maji, and Mukherjee [14] introduced an inductive approach that characterizes a lower bound on the insecurity $C_{n}\left(X_{0}\right)$ of any such coin-tossing protocol. Furthermore, their approach is constructive, i.e., it constructs a coin-tossing protocol such that its insecurity is, at most, $C_{n}\left(X_{0}\right)$. Surprisingly, these secure coin-tossing protocols are more secure than the folklore constructions widely believed to be optimal earlier.
2. Algebraized version. Section 4 presents an algebraic version of the proof technique mentioned above, as introduced by [16,30]. This proof technique sacrifices a small constant factor on the lower bound on insecurity. However, the algebraized proof technique extends to more complicated information-theoretic models where parties have access to oracles. These lower bounds to insecurity in complex relativized settings translate into black-box separation results [31,32] settling several long-standing open problems.
3. Connection to isoperimetric inequalities. Section 5 establishes a connection between the security of optimal coin-tossing protocols in the information-theoretic model and isoperimetric inequalities in product spaces over large alphabets. Isoperimetric inequalities in product spaces of large alphabets are known to be not sufficiently well-behaved. The cryptographic perspective into isoperimetric inequalities makes a case for new “symmetrized” versions of these isoperimetric inequalities. For example, the initial results of [29] demonstrate that these symmetrized isoperimetric inequalities are significantly more well-behaved.

## 2. Preliminaries and Model

### 2.1. System Specification

Following Ben-Or and Linial [1,2], the survey considers the standard *n*-processor coin-tossing protocols in the *full information* setting, i.e., the processors are computationally unbounded and send their messages over a common broadcast channel. The coin-tossing protocol proceeds in *rounds*, where a subset of the processors broadcast their messages in that round, and this subset of processors possibly depends on the messages broadcast in the previous rounds. In a *t-turn* coin-tossing protocol, each processor sends (at most) *t* messages during the entire protocol execution. When the protocol completes, all processors agree on a common output, their collective coin. Intuitively, a collective coin-tossing protocol upgrades the local private randomness of multiple parties into their shared randomness.

### 2.2. Extensions

The base system mentioned above is the *information-theoretic plain model*. The survey also encompasses this base system’s extension with oracles (for example, a *random oracle*) and other ideal secure computation functionalities (for example, *secure function evaluation* functionalities). These extensions enable studying the complexity of secure coin-tossing relative to various hardness of computation assumptions and the complexity of performing other secure computations. For example, the *random oracle model* provides random oracle access to the parties. That is a random ${\left\{0,1\right\}}^{n}\to {\left\{0,1\right\}}^{n}$ function, where *n* represents the bit-length of the input to the random oracle. Intuitively, a random oracle answers old queries consistently and new queries uniformly and independently at random from the set ${\left\{0,1\right\}}^{n}$. The *f-hybrid model* provides parties access to the ideal *f*-functionality.

### 2.3. Adversary and Security Model

A Byzantine adversary with *corruption threshold k* may corrupt up to *k* processors statically (i.e., the adversary decides which processors to corrupt before the protocol execution starts), or adaptively (i.e., the adversary corrupts processors depending on the protocol evolution). A *strong* adaptive adversary [33] can observe a processor’s message before corrupting it. The adversary controls all corrupted processors’ messages and is *rushing*; that is, all honest processors in a particular round broadcast their message first, and then the adversary determines the messages of the corrupted processors for that round. The adversary may choose to abort the protocol execution prematurely.

The survey considers both *security with abort* and *security with guaranteed output delivery.* Intuitively, if the adversary aborts, security with abort permits the protocol to abort without providing any output to the honest processors. On the other hand, the significantly stringent security notion of guaranteed output delivery insists that the honest processors receive output even if the adversary aborts. A coin-tossing protocol is *ε-insecure* if the adversary can change the honest processors’ output distribution by, at most, ε in the total variation distance (or, equivalently, statistical distance).

### 2.4. Notations and Terminology

In the sequel, the common output of a coin-tossing protocol is 0 or 1. Intuitively, 1 represents heads, and 0 represents tails. The expected output of a coin-tossing protocol represents the probability of its output being heads. A *bias-X* coin-tossing protocol is an interactive protocol whose expected (common) output is X∈[0,1].

This survey considers *r*-round coin-tossing protocols. The *transcript exposure filtration* reveals the messages of every round sequentially. The partial transcript of a protocol after *i* rounds is $\left(T_{1},\;\ldots ,\;T_{i}\right)$, i.e., the concatenation of the messages broadcast in rounds 1,…,i. Conditioned on the transcript, being $T_{1},\;\ldots ,\;T_{i}$, the random variable $X_{i}$ represents the expected output of the protocol. Note that $X_{r}\in \left\{0,1\right\}$, that is, all processors agree on the output at the end of the protocol. Furthermore, the random variable $X_{0}$ represents the expected output of the protocol before the protocol began, that is, $X_{0}=X$ for a bias-*X* coin-tossing protocol. Observe that $\left(X_{0},X_{1},\;\ldots ,\;X_{r}\right)$ is a *martingale* w.r.t. the transcript exposure filtration.

### 2.5. Coin-Tossing Protocols as Trees

One can represent coin-tossing protocols equivalently as labeled trees. This tree representation helps develop a combinatorial intuition for the results, and enables a succinct and intuitive (yet, precise) presentation of the primary technical ideas. Every node in the tree corresponds to a partial transcript $\left(T_{1},\;\ldots ,\;T_{i}\right)$. For a node *v* in the tree, T(v) represents its corresponding partial transcript. If a node *u* represents the transcript $\left(T_{1},\;\ldots ,\;T_{i-1}\right)$ and a node *v* represents the transcript $(T_{1},\;\ldots ,\;T_{i}),$ then *u* is the *parent* of *v*. The *root* of the tree corresponds to the empty transcript ∅, and the leaves correspond to the complete transcripts of the protocol. The label on the edge (u,v) is the value of the random variable $T_{i}$ (that is, the message sent in round *i*).

The *color* of a node *v*, represented by X(v)∈[0,1], is the expected output of the protocol conditioned on the partial transcript being T(v). Therefore, leaves have color 0 or 1, and the root has color *X* in a bias-*X* coin-tossing protocol. The coin-tossing protocol Π(v) represents the bias-X(v) coin-tossing protocol associated with subtree rooted at *v*.

**Figure 1:**
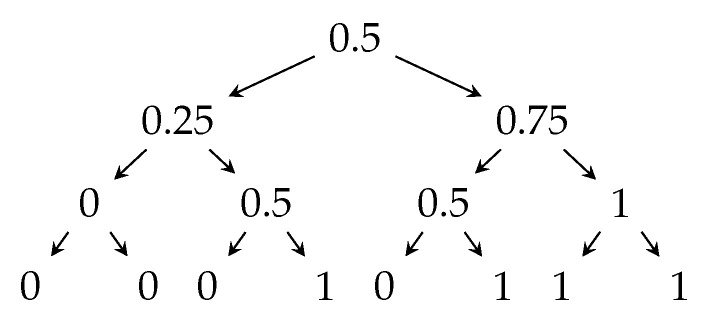
Transcript tree for the majority protocol, where n=3.

For illustrative purposes, Figure 1 presents the tree representation of the “majority protocol” for n=3 processors. In round i∈{1,2,…,n}, processor *i* broadcasts an independent uniformly random bit. The collective output b∈{0,1} after *n* bits have been broadcast is the majority of these bits. The edge going left corresponds to the broadcast message being 0, and the edge going right corresponds to the broadcast message being 1. The nodes are labeled by their color.

## 3. Optimal Coin-Tossing Protocols: A Geometric Approach

This section introduces the original combinatorial technique of Khorasgani, Maji, and Mukherjee [14] for characterizing the “most secure” coin-tossing protocol.

### 3.1. A Representative Motivating Application

Consider a distributed collective coin-tossing protocol for *n* processors, where a processor *i* broadcasts its message in round *i*. At the end of the protocol, all processors reconstruct the common output from the public transcript. When all processors are honest, the probability of the final output being 1 is $X_{0}$ and the probability of the final output being 0 is $1-X_{0}$, i.e., the final output is a *bias-$X_{0}$ coin*. Suppose there is an adversary who can (adaptively) choose to *restart* one of the processors after seeing her message (i.e., the *strong adaptive* corruptions model introduced by Goldwasser, Kalai, and Park [33]); otherwise her presence is innocuous. Our objective is to design bias-$X_{0}$ coin-tossing protocols, such that the adversary cannot significantly change the distribution of the final output.

In summary, we consider single-turn collective coin-tossing protocols where only one processor broadcasts every round. We consider security with abortion against an adversary that is strong [33] and adaptive. The adversary can perform a soft attack where it may restart a processor if it does not like its message.

*The Majority Protocol.* Against computationally unbounded adversaries, (essentially) the only known protocol is the well-known majority protocol [34,35,36,37] for $X_{0}=1/2$. The majority protocol requests one uniformly random bit from each processor and the final output is the majority of these *n* bits. An adversary can alter the expected output by $1/\sqrt{2\pi n}$ (more specifically, the fractional weight of the central binomial coefficient), i.e., the majority protocol is $1/\sqrt{2\pi n}$-insecure. More generally, one considers *threshold protocols*, where the collective output is 1 if and only if the total number of broadcast bits is more than a fixed threshold.

**Figure 2:**
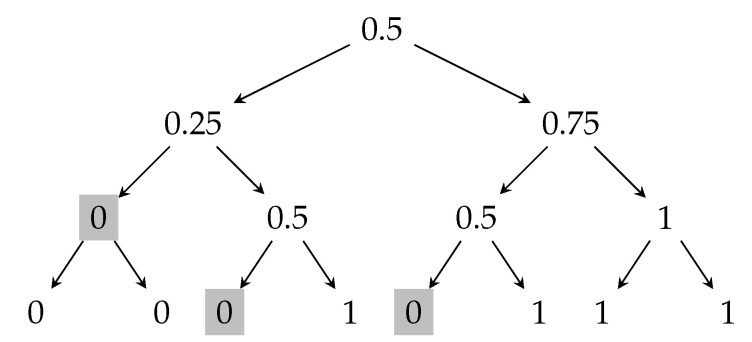
Transcript tree for the majority protocol, where n=3. The shaded nodes represent the partial transcripts where the adversary restarts the processor that broadcast the last message in the protocol.

Figure 2 shows the optimal attack on the majority protocol for n=3 that increases the expected output of the protocol. The shaded nodes in the tree represents the partial transcripts where the adversary intervenes and restarts the last processor that broadcast its message. The insecurity of this protocol is nn/2·2−n=0.1875. Figure 8, as a consequence of the recent works [14,38], presents a protocol that has higher security than this majority protocol.

Towards this objective, first, the survey summarizes the new proof technique introduced by Khorasgani, Maji, and Mukherjee [14] that yields a two-approximation to the optimal solution of the motivating problem above (Section 3.4 summarizes this proof technique). Section 3.6 includes empirical results summarizing conjectured constructions that have higher security than the threshold protocols.

### 3.2. Martingale Problem Statement

Given a complete transcript, let τ∈{1,2,…,n,∞} represent the round where the adversary intervenes. Observe that, by restarting the processor at τ∈{1,2,…,n}, the adversary changes the expected output of the protocol from $X_{\tau }$ to $X_{\tau -1}$. Therefore, the change in the expected output of the protocol is $X_{\tau -1}-X_{\tau }.$ The intervention strategy of an adversary is equivalently represented as a stopping time τ:Ω→{1,2,…,n,∞}, where Ω is the set of all complete transcripts of the coin-tossing protocol. If the associated stopping time for a complete transcript is *∞*, then the adversary does not intervene during the generation of that complete transcript. The increase in the expected output corresponding to this adversarial strategy τ is equal to

$$E[X_{\tau -1}-X_{\tau }].$$

For the simplicity of presenting the primary technical ideas, it is instructive to consider a related, albeit slightly different, *score function*

$$E[|X_{\tau }-X_{\tau -1}|].$$

The inspiration of the approach introduced by Khorasgani, Maji, Mukherjee [14] is best motivated using a two-player game between, namely, the *martingale designer* and the *adversary*. Fix *n* and $X_{0}$. The martingale designer presents a martingale $X=(X_{0},X_{1},\;\ldots ,\;X_{n})$ (w.r.t. to the transcript exposure filtration) to the adversary and the adversary finds a stopping time τ that maximizes the score function.

$$E[|X_{\tau }-X_{\tau -1}|]$$

Intuitively, the adversary demonstrates the most severe *susceptibility* of the martingale by presenting the corresponding stopping time τ as a witness. The stopping time witnessing the highest susceptibility shall translate into appropriate adversarial strategies. The martingale designer’s objective is to design martingales that have less susceptibility. Khorasgani et al. [14] introduce a geometric approach to inductively provide tight bounds on the least susceptibility of martingales for all n≥1 and $X_{0}\in \left[0,1\right]$, that is, the following quantity.

$$C_{n}\left(X_{0}\right)\underset{X}{\inf}\;\underset{\tau }{\sup}\;E[|X_{\tau }-X_{\tau -1}|]$$

Similar to [10], this precise study of $C_{n}\left(X_{0}\right)$, for general $X_{0}\in \left[0,1\right]$, is motivated by natural applications in discrete process control as illustrated by the representative motivating problem.

### 3.3. Prior Approaches to the General Martingale Problem

Azuma–Hoeffding inequality [39,40] states that, if $|X_{i}-X_{i-1}|=o\left(1/\sqrt{n}\right)$, for all i∈{1,…,n}, then, essentially, $|X_{n}-X_{0}|=o\left(1\right)$ with probability 1. That is, the final information $X_{n}$ remains close to the a priori information $X_{0}$. However, in our problem statement, we have $X_{n}\in \left\{0,1\right\}$. In particular, this constraint implies that the final information $X_{n}$ is significantly different from the a priori information $X_{0}$. So, the initial constraint “for all i∈{1,…,n} we have $|X_{i}-X_{i-1}|=o\left(1/\sqrt{n}\right)$” must be violated. What is the probability of this violation?

For $X_{0}=1/2$, Cleve and Impagliazzo [10] proved that there exists a round *i* such that $|X_{i}-X_{i-1}|\geq \frac{1}{32\sqrt{n}}$ with probability 1/5. We emphasize that the round *i* is a random variable and not a constant. However, the definition of the “big jump” and the “probability to encounter big jumps” are both exponentially small functions of $X_{0}$. So, the approach of Cleve and Impagliazzo is only applicable to constant $X_{0}\in \left(0,1\right)$. Recently, in an independent work, Beimel et al. [41] demonstrate an identical bound for *weak martingales* (that have some additional properties), which is used to model multi-party coin-tossing protocols.

For the upper-bound, on the other hand, Doob’s martingale, corresponding to the majority protocol, is the only known martingale for $X_{0}=1/2$ with a small *maximum susceptibility*. In general, to achieve arbitrary $X_{0}\in \left[0,1\right]$, one considers coin-tossing protocols, where the output is 1 if the total number of heads in *n* uniformly random coins surpasses an appropriate threshold.

### 3.4. Inductive Approach

This section presents a high-level overview of the inductive strategy to characterizing optimal coin-tossing protocols. In the sequel, we shall assume that we are working with discrete-time martingales $\left(X_{0},X_{1},\;\ldots ,\;X_{n}\right)$ such that $X_{n}\in \left\{0,1\right\}$.

Given a martingale $\left(X_{0},\;\ldots ,\;X_{n}\right)$, its *susceptibility* is represented by the following quantity

$$\underset{\mathrm{stopping}\;\mathrm{time}\;\tau }{\sup}E[|X_{\tau }-X_{\tau -1}|]$$

Intuitively, if a martingale has high susceptibility, then it has a stopping time, such that the gap in the martingale while encountering the stopping time is large. Our objective is to characterize the *least susceptibility* that a martingale $\left(X_{0},\;\ldots ,\;X_{n}\right)$ can achieve. More formally, given *n* and $X_{0}$, characterize

$$C_{n}\left(X_{0}\right):=\underset{\left(X_{0},\;\ldots ,\;X_{n}\right)}{\inf}\underset{\mathrm{stopping}\;\mathrm{time}\;\tau }{\sup}E[|X_{\tau }-X_{\tau -1}|].$$

The approach proceeds by induction on *n* to exactly characterize the curve $C_{n}\left(X\right)$, and our argument naturally constructs the best martingale that achieves $C_{n}\left(X_{0}\right)$.

**Figure 3:**
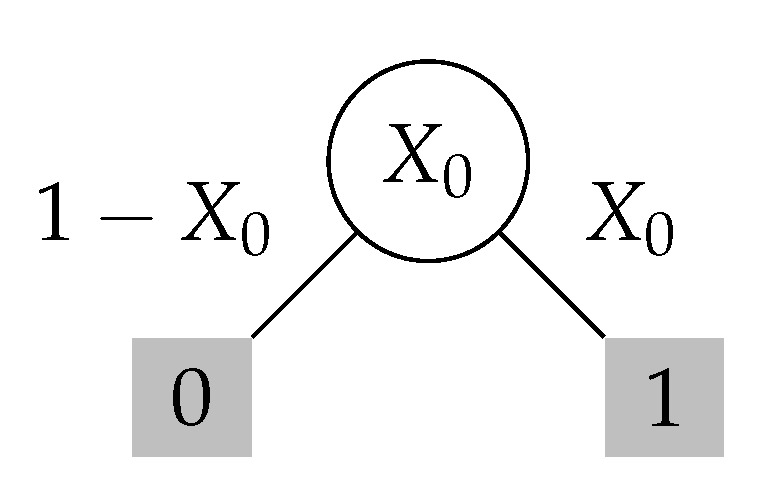
Base case for the inductive approach. Note $C_{1}\left(X_{0}\right)=\inf_{\left(X_{0},X_{1}\right)}\sup_{\tau }E[|X_{\tau }-X_{\tau -1}|]$. The optimal stopping time is shaded and its score is $X_{0}\cdot |1-X_{0}|+\left(1-X_{0}\right)\cdot \left|0-X_{0}\right|=2X_{0}\left(1-X_{0}\right)$.

1. Base case. Note that the base case is $C_{1}\left(X\right)=2X\left(1-X\right)$ (see Figure 3 for this argument).
2. Inductive step. Given the curve $C_{n-1}\left(X\right)$, one identifies a *geometric transformationT* (see Figure fig:transform-def) that defines the curve $C_{n}\left(X\right)$ from the curve $C_{n-1}\left(X\right)$.
  Furthermore, for any n≥1, there exist martingales such that its susceptibility is exactly $C_{n}\left(X_{0}\right)$.

We shall prove the following technical result in this section.

**Theorem** **1.** *Fix any $X_{0}\in \left(0,1\right)$ and n∈ℕ. Let $X=(X_{0},X_{1},\;\ldots ,\;X_{n})$ be a martingale, such that $X_{n}\in \left\{0,1\right\}$. There exists a stopping time τ in such that*

$$E[|X_{\tau }-X_{\tau -1}|]\geq C_{n}\left(X\right).$$

Furthermore, for all n∈ℕ and $X_{0}\in \left(0,1\right)$, there exists a martingale $X^{*}=\left(X_{0},X_{1}^{*},\;\ldots ,\;X_{n}^{*}\right)$ such that $X_{n}^{*}\in \left\{0,1\right\}$ and, for all stopping times τ, we have

$$E[|X_{\tau }^{*}-X_{\tau -1}^{*}|]=C_{n}\left(X_{0}\right).$$

Base Case of n=1

Refer to Figure 3 for the following discussion. For a martingale $\left(X_{0},X_{1}\right)$ of depth n=1, we have $X_{1}\in \left\{0,1\right\}$. Thus, without loss of generality, we assume that $E_{1}$ takes only two values. Then, it is easy to verify that the max score is always equal to $2X_{0}\left(1-X_{0}\right)$. This score is witnessed by the stopping time τ=1. So, we conclude that $C_{1}\left(X_{0}\right)=2X_{0}\left(1-X_{0}\right).$

Inductive Step: n=2 (For Intuition).

**Figure 4:**
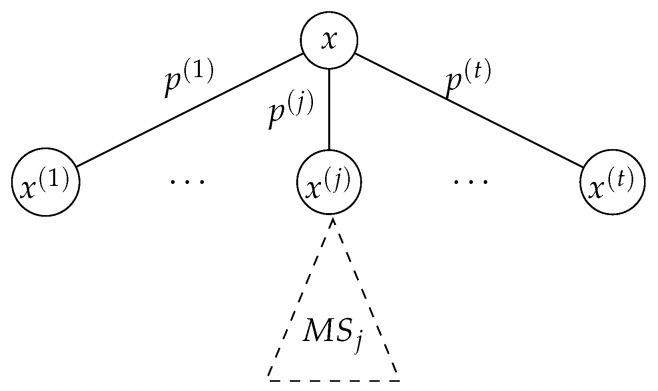
The inductive step of the combinatorial proof. ${MS}_{j}$ represents the max-score of the sub-tree of depth n−1 which is rooted at $x^{\left(j\right)}$. For simplicity, the subtree of $x^{\left(j\right)}$ is only shown here.

Suppose that the root $X_{0}=x$ in the corresponding martingale tree has *t* children with values $x^{\left(1\right)},x^{\left(2\right)},\ldots ,x^{\left(t\right)}$, and the probability of choosing the *j*-th child is $p^{\left(j\right)}$, where j∈{1,…,t} (see Figure 4).

Given a martingale $\left(X_{0},X_{1},X_{2}\right)$, the adversary’s objective is to find the stopping time τ that maximizes the score $E|X_{\tau }-X_{\tau -1}|$. If the adversary chooses to stop at τ=0, then the score $E[|X_{\tau }-X_{\tau -1}|]=0$, which is not a good strategy. So, for each *j*, the adversary chooses whether to stop at the child $x^{\left(j\right)}$, or defer the attack to a stopping time in the sub-tree rooted at $x^{\left(j\right)}$. The adversary chooses the stopping time based on which of these two strategies yield a better score. If the adversary stops the martingale at child *j*, then the contribution of this decision to the score is $p^{\left(j\right)}\cdot \left|x^{\left(j\right)}-x\right|$. On the other hand, if she does not stop at child *j*, then the contribution from the sub-tree is guaranteed to be $p^{\left(j\right)}\cdot MS_{j}\geq p^{\left(j\right)}\cdot C_{1}\left(x^{\left(j\right)}\right)$. Overall, from the *j*-th child, an adversary obtains a score that is at least $p^{\left(j\right)}\cdot \max\left\{|x^{\left(j\right)}-x|,C_{1}\left(x^{\left(j\right)}\right)\right\}$.

**Figure 5:**
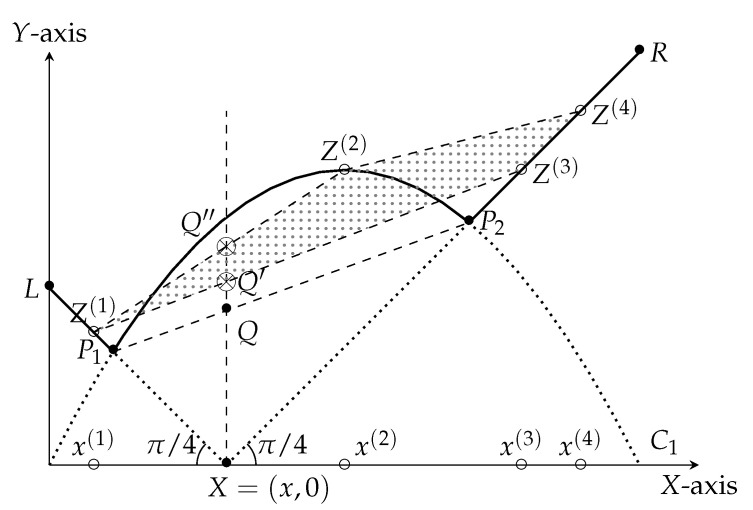
Intuitive summary of the inductive step for n=2.

Let $h^{\left(j\right)}:=\max\left\{|x^{\left(j\right)}-x|,C_{1}\left(x^{\left(j\right)}\right)\right\}$. We represent the points $Z^{\left(j\right)}=\left(x^{\left(j\right)},h^{\left(j\right)}\right)$ in a two dimensional plane. Then, clearly, all these points lie on the solid curve defined by $\max\left\{|X-x|,C_{1}\left(X\right)\right\}$—see Figure 5.

Since (X,E) is a martingale, we have $x=\sum_{j=1}^{t}p^{\left(j\right)}x^{\left(j\right)}$ and the adversary’s strategy for finding $\tau_{\max}$ gives us $\lambda =\sum_{j=1}^{t}p^{\left(j\right)}h^{\left(j\right)}$. This observation implies that the coordinate $\left(x,\lambda \right)=\sum_{j=1}^{t}p^{\left(j\right)}\cdot Z^{\left(j\right)}$. So, the point in the plane giving the adversary the maximum score for a tree of depth n=2 with bias $X_{0}=x$ lies in the *intersection* of the convex hull of the points $Z^{\left(1\right)},\;\ldots ,\;Z^{\left(t\right)}$, and the line X=x. Let us consider the martingale defined in Figure 5 as a concrete example. Here t=4, and the points $Z^{\left(1\right)},Z^{\left(2\right)},Z^{\left(3\right)},Z^{\left(4\right)}$ lie on $\max\left\{|X-x|,C_{1}\left(X\right)\right\}$. The martingale designer specifies the probabilities $p^{\left(1\right)},p^{\left(2\right)},p^{\left(3\right)}$, and $p^{\left(4\right)}$, such that $p^{\left(1\right)}x^{\left(1\right)}+\cdots +p^{\left(4\right)}x^{\left(4\right)}=x$. These probabilities are not represented in Figure 5. Note that the point $\left(p^{\left(1\right)}x^{\left(1\right)}+\cdots +p^{\left(4\right)}x^{\left(4\right)},p^{\left(1\right)}h^{\left(1\right)}+\cdots +p^{\left(4\right)}h^{\left(4\right)}\right)$ representing the score of the adversary is the point $p^{\left(1\right)}Z^{\left(1\right)}+\cdots +p^{\left(4\right)}Z^{\left(4\right)}$. This point lies inside the convex hull of the points $Z^{\left(1\right)},\ldots ,Z^{\left(4\right)}$ and on the line $X=p^{\left(1\right)}x^{\left(1\right)}+\cdots +p^{\left(4\right)}x^{\left(4\right)}=x$. The exact location depends on $p^{\left(1\right)},\ldots ,p^{\left(4\right)}$.

Point $Q^{\prime }$ is the point with minimum height. Observe that the height of the point $Q^{\prime }$ is at least the height of the point *Q*. So, in any martingale, the adversary shall find a stopping time that scores more than (the height of) the point *Q*.

On the other hand, the martingale designer’s objective is to reduce the score that an adversary can achieve. So, the martingale designer chooses t=2, and the two points $Z^{\left(1\right)}=P_{1}$ and $Z^{\left(2\right)}=P_{2}$ to construct the optimum martingale. We apply this method for each x∈[0,1] to find the corresponding point *Q*; that is, the *locus of the point Q*, for x∈[0,1], which yields the curve $C_{2}\left(X=x\right)$.

Observe that the height of the point *Q* is the *harmonic-mean* of the heights of the points $P_{1}$ and $P_{2}$. This observation follows from elementary geometric facts. Let $h_{1}$ represent the height of the point $P_{1}$, and $h_{2}$ represent the height of the point $P_{2}$. Observe that the distance of $x-x_{S}\left(x\right)=h_{1}$ (because the line $\ell_{1}$ has slope π−π/4). Similarly, the distance of $x_{L}\left(x\right)-x=h_{2}$ (because the line $\ell_{2}$ has slope π/4). So, using properties of similar triangles, the height of *Q* turns out to be

$$h_{1}+\frac{h_{1}}{h_{1}+h_{2}}\cdot \left(h_{2}-h_{1}\right)=\frac{2h_{1}h_{2}}{h_{1}+h_{2}}.$$

**Figure 6:**
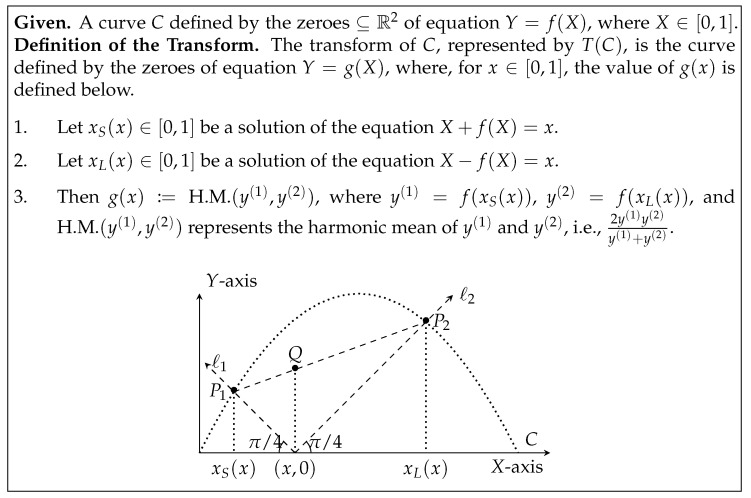
Definition of transform of a curve *C*, represented by T(C). The locus of the point *Q* (in the right figure) defines the curve T(C).

This property inspires the definition of the geometric transformation *T*, see Figure 6. Applying *T* on the curve $C_{1}\left(X\right)$ yields the curve $C_{2}\left(X\right)$. All bias-*X* (n=2) processor coin-tossing protocols are $C_{n}\left(X\right)$-insecure.

Furthermore, there exists a coin-tossing protocol that achieves this insecurity bound.

General Inductive Step: n≥2

Note that a similar approach works for general n=d≥2. Fix $X_{0}$ and n=d≥2. We assume that the adversary can compute $C_{d-1}\left(X_{1}\right)$, for any $X_{1}\in \left[0,1\right]$.

Suppose the root in the corresponding martingale tree has *t* children with values $x^{\left(1\right)},x^{\left(2\right)},\;\ldots ,\;x^{\left(t\right)}$, and the probability of choosing the *j*-th child is $p^{\left(j\right)}$ (see Figure 4). Let $\left(X^{\left(j\right)},E^{\left(j\right)}\right)$ represent the martingale associated with the sub-tree rooted at $x^{\left(j\right)}$.

For any j∈{1,…,t}, the adversary can choose to stop at the child *j*. This decision will contribute $|x^{\left(j\right)}-x|$ to the score with weight $p^{\left(j\right)}$. On the other hand, if she defers the attack to the subtree rooted at $x^{\left(j\right)}$, she will get at least a contribution of (at least) $C_{n-1}\left(x^{\left(j\right)}\right)$, with weight $p^{\left(j\right)}$. Therefore, the adversary can obtain the following contribution to her score

$$p^{\left(j\right)}\max\left\{|x^{\left(j\right)}-x|,C_{d-1}\left(x^{\left(j\right)}\right)\right\}$$

**Figure 7:**
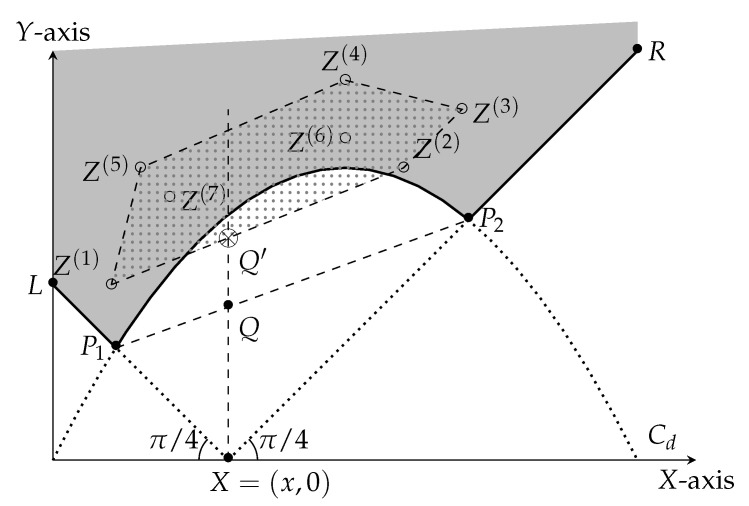
Intuitive summary of the inductive argument. Our objective is to pick the set of points $\left\{Z^{\left(1\right)},Z^{\left(2\right)}\ldots \right\}$ in the gray region to minimize the length of the intercept $XQ^{\prime }$ of their (lower) convex hull on the line X=x. Clearly, the unique optimal solution corresponds to including both $P_{1}$ and $P_{2}$ in the set.

Similar to the case of n=2, we define the points $Z^{\left(1\right)},\;\ldots ,\;Z^{\left(t\right)}$. For n>2, however, there is one difference from the n=2 case. The point $Z^{\left(j\right)}$ need not *lie on the solid curve*, but it can lie on or above it, i.e., they lie in the gray area of Figure 7. This phenomenon is attributable to a suboptimal martingale designer, producing martingales with suboptimal scores, i.e., *strictly above* the solid curve. For n=1, it happens to be the case that there is (effectively) only one martingale that the martingale designer can design (the optimal tree). The adversary obtains a score that is at least the height of the point $Q^{\prime }$, which is at least the height of *Q*. On the other hand, the martingale designer can choose t=2, and $Z^{\left(1\right)}=P_{1}$ and $Z^{\left(2\right)}=P_{2}$ to define the optimum martingale. Again, the locus of point *Q* is defined by the curve $T(C_{d-1})$.

Conclusion

So, by induction, we have proved that $C_{n}\left(X\right)=T^{n-1}\left(C_{1}\left(X\right)\right)$. Additionally, note that, during induction, in the optimum martingale, we always have $|x^{\left(0\right)}-x|=C_{n-1}\left(x^{\left(0\right)}\right)$ and $|x^{\left(1\right)}-x|=C_{n-1}\left(x^{\left(1\right)}\right)$. Intuitively, the decision to stop at $x^{\left(j\right)}$ or continue to the subtree rooted at $x^{\left(j\right)}$ has identical consequence. So, by induction, *all stopping times* in the optimum martingale have score $C_{n}\left(x\right)$.

A close-form characterization of $C_{n}\left(X\right)$ using elementary functions seems challenging. Khorasgani et al. [14] proved the following upper and lower bounds.

$$\min\left\{\frac{2}{\sqrt{n+3}}\cdot \sqrt{X(1-X)},2X,2-2X\right\}\geq C_{n}\left(X\right)\geq \sqrt{\frac{2}{n-1/2}}\cdot X\left(1-X\right).$$

### 3.5. Related Work: Multiple Corruptions

Another line of research characterizes the minimum number of corruptions *t* that suffices to change the expected output of the coin-tossing protocol by a constant. The presentation below, for simplicity, ignores polylogarithmic factors in the asymptotic notation. The authors in [42] proved that a Byzantine adversary can adaptively corrupt $t=\tilde{O}\left(\sqrt{n}\right)$ processors in any *n*-processor single-turn protocol, where every processor broadcasts one-bit messages, to change the expected output of the protocol by a constant. Subsequently, [33,43] generalized this result to the case where the processors broadcast arbitrary-length messages. Recently, in a breakthrough result, Haitner and Karidi-Heller [44] extended this result to *multi-turn* coin-tossing protocols, i.e., a processor may send messages in multiple rounds. Essentially, these results imply that the majority protocol (more generally, the threshold protocols) are qualitatively optimal. However, the characterization of the most secure coin-tossing protocols remains open.

A prominent model in distribution computing considers the following adversarial model for coin-tossing protocols. A strong adversary can adaptively corrupt (up to) *t* processors and the messages of all corrupted processors are erased. Aspnes [22,23] uses an inductive approach to characterize the robustness of such coin-tossing protocols. This approach also uses a geometric approach to perform induction on *t*, the number of corruptions that the adversary makes, to account for (a) the maximum increase in the expected output of the coin-tossing protocol and (b) the maximum decrease in the expected output of the coin-tossing protocols. [22,23] proves that $t=O(\sqrt{n})$ suffices to change the expected output of an *n*-processor coin-tossing protocol by a constant. However, this inductive approach is non-constructive because the recursion does not characterize the evolution of the martingale corresponding to the most secure coin-tossing protocol.

### 3.6. Experimental Results

**Figure 8:**
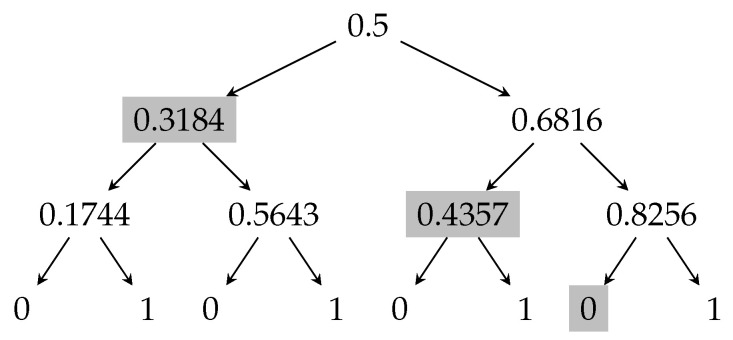
Transcript tree for the optimal protocol corresponding to the recursion $A_{1}\left(X\right)X\left(1-X\right)$ and $A_{n}=T^{n-1}\left(A_{1}\right)$, where n=3. The shaded partial transcripts represents the attack that increases the expected output by 0.1362, which is also the maximum insecurity of this coin-tossing protocol.

The presentation above considers the case where the stopping time representing an adversarial strategy is τ:Ω→{1,2,…,n} (where Ω represents the set of all complete transcripts), and the score of a stopping time is $E[|X_{\tau }-X_{\tau -1}|].$ Khorasgani, Maji, Mehta, Mukherjee, and Wang [14,38] study a related recursion. In this recursion, the stopping time is τ:Ω→{1,2,…,n,∞}. However, the stopping times are restricted as follows. Given a partial transcript *u*, if the adversary has the following choices: (1) Do not abort for any child of *u*; (2) Abort at all children *v*, such that X(v) (i.e., the expected output conditioned on *v*) is at least a particular threshold; (3) Abort at all children *v* such that X(v) is at most a particular threshold. The optimal score for such restricted stopping times is represented by $A_{n}\left(X\right)$. The authors in [38] construct an algorithm with running time poly(n,1/δ) for computing $A_{n}\;:=T^{n-1}\left(A_{1}\right),$ where $A_{1}\left(X\right)=X\left(1-X\right)$ with (at most) nδ error. We highlight that the geometric transformation T(·) is identical to the one presented in Section 3.4. However, the base cases are different; $A_{1}\left(X\right)=X\left(1-X\right)$, but $C_{1}\left(X\right)=2X\left(1-X\right)$. Now, consider the optimal protocol corresponding to this recursion. For example, Figure 8 shows the martingale corresponding to $X_{0}=1/2$ and n=3. The optimal attack that increases the expected output is represented by the shaded nodes. Restarting the last processor broadcasting the message resulting in a shaded partial transcript increases the output by 0.1362, which is significantly less than 0.1865, the insecurity of the majority protocol from Figure 2.

**Figure 9:**
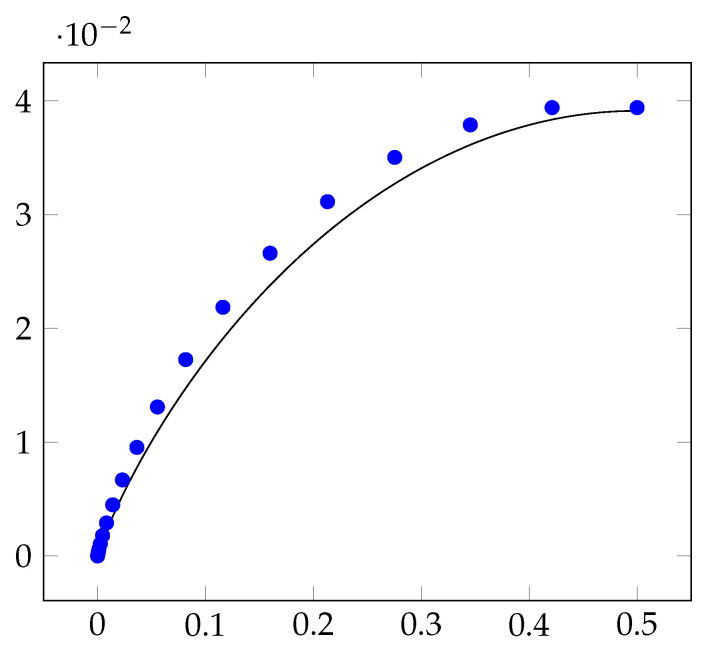
The X-axis represents the bias of the coin-tossing protocols and the Y-axis represents the insecurity of the protocols. For n=101, the blue marks denote the insecurity of bias-*X* coin-tossing protocols that are implementable using a threshold protocol. The black curve represents the insecurity of our new protocols. A coin-tossing protocol with lower insecurity is more desirable.

Experimentally, we implement our protocol and show that the insecurity of our protocol is observably smaller than the insecurity of threshold protocols. As a representative example, Figure 9 plots the insecurity of our new protocol, for n=101 processors and X∈[0,1/2] with accuracy parameter $\delta =10^{-6}$. This demonstrates the insecurity of bias-*X* coin-tossing protocols, where X∈(1/2,1], is identical to the insecurity of bias-(1−X) coin-tossing protocols. So, it suffices to consider bias-*X* protocols, where X∈[0,1/2].

Figure 9 also plots the insecurity of all bias-*X* coin-tossing protocols that can be implemented using a threshold protocol. Note that the insecurity of our protocol is less than the insecurity of threshold protocol. This reduction in insecurity is prominent, especially when X∈(0,1/2) is simultaneously far from 0 and 1/2.

**Figure 10:**
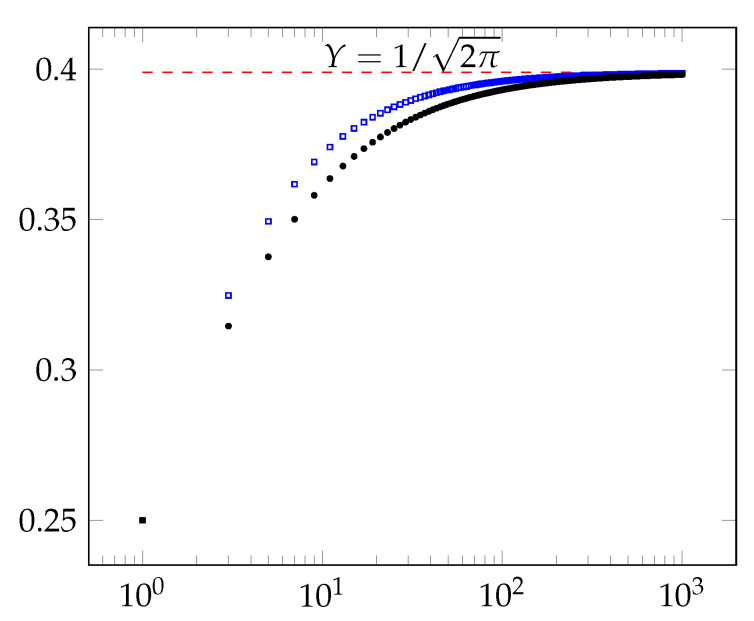
For n∈{1,3,…,1001}, the blue squares show the plot of $\sqrt{n}$-times the insecurity of the majority coin-tossing protocol. The red dashed line shows the limit of the insecurity of majority protocol using Stirling’s approximation, when n→∞. The black dots show the plot of $\sqrt{n}$-times the security of our new optimal protocols. The graph uses log scale on the X-axis.

Finally, our experiments uncover an exciting phenomenon. As Figure 10 indicates, our experimental results show that the insecurity of our protocols for X=1/2 tends towards the insecurity of the majority protocol, as *n* tends to infinity. This experiment lends support to the conjecture that the majority protocol is the optimal secure coin-tossing protocol as n→∞. However, for every finite *n* and X∈(0,1/2), there are more secure protocols than the threshold protocols.

## 4. Hardness of Computation Relative to Oracles

This section considers two-processor one-message-per-round coin-tossing protocols with guaranteed output delivery. The first processor, say, Alice sends messages in rounds {1,3,…}, and the second processor (Bob) sends messages in rounds {2,4,…}. This coin-tossing protocol has *n*-rounds, and Alice has [n/2] turns and Bob has [n/2] turns. A Byzantine adversarial processor may abort prematurely during the protocol execution; however, the honest processor still has to output a coin. This coin-tossing protocol is the well-known *fair coin-tossing protocol* [45]. A fair coin-tossing protocol is *ε-unfair* if it is ε-insecure.

### 4.1. Summary of Relevant Work

#### 4.1.1. Impagliazzo’s Worlds

Among the several potential approaches to summarize the state-of-the-art in this field, the author prefers positioning the results in Impagliazzo’s multiverse [46] (refer to Table 1). The sequel considers three of the five Impagliazzo’s multiverses, which are most relevant to the discussion. In Pessiland, every efficient function is efficiently invertible, i.e., one-way functions do not exist [47]. One-way functions exist in Minicrypt and allow for private-key cryptographic primitives, such as pseudorandom generators [48,49,50], pseudorandom functions [51,52], pseudorandom permutations [53], statistically binding commitment [54], statistically hiding commitment [55,56], zero-knowledge proofs [57], and digital signatures [58,59]. However, public-key cryptography, as with public-key encryption, key-agreement, and general secure computation, are not possible in Minicrypt [31,60,61,62]. The secure constructions of these primitives lie in Cryptomania. One should imagine Cryptomania as a conglomeration of infinitely many hardness of computation assumptions that are *not* securely attainable based solely on the existence of one-way functions. Intuitively, Minicrypt enables fast cryptography, while Cryptomania supports more sophisticated, yet comparatively slower, cryptography.

#### 4.1.2. Relevant Results

In the information-theoretic setting, in any protocol, one of the processors can always force an output with certainty (using attacks in two-player zero-sum games, or games against nature [68]). However, one can emulate this attack relying on a significantly weaker assumption. For instance, if one-way functions do not exist [47] then Berman, Haitner, Omri, and Tentes [65,66] rely on rejection sampling techniques to construct a Byzantine adversary that forces an output with (near) certainty. (Intuitively, the assumption that “one-way functions do not exist” is slightly weaker than the assumption that “NP ⊆ BPP,” because the latter implies the former. However, these two assumptions are extremely similar in nature as well. The former assumption, roughly states that every efficient function is efficiently invertible on an average. The latter assumption, on the other hand, states that every efficient function is efficiently invertible for every element in the range. For example, the constant-unfairness of any coin-tossing protocol, based on the assumption that NP ⊆ BPP, is implied by [69].) That is, in summary, if one-way functions do not exist, then every coin-tossing protocol is Ω(1)-unfair. If one restricts to attacks where parties are honest-but-curious but may abort during the protocol execution, referred to as *fail-stop adversaries*, then, if one-way functions do not exist, every coin-tossing protocol is $\Omega (1/\sqrt{n})$-unfair [10,14].

On the other hand, if one-way functions exist, then (using commitments and zero-knowledge proofs) there are protocols that are $O(1/\sqrt{n})$-insecure [34,35,36,37], that is, the adversary can change the honest processor’s output distribution by at most $O(1/\sqrt{n})$ (in the statistical distance). In a ground-breaking result, Moran, Naor, and Segev [67] presented the first fair coin-tossing protocol that is only O(1/n)-unfair based on the existence of (unfair) secure protocols for oblivious transfer functionality. Cleve [37] proved that Ω(1/n)-unfairness is unavoidable; hence, the protocol by Moran, Naor, and Segev is “optimal.”

**Recent Advances.** A recent work [16] proves that any fair coin-tossing protocol using one-way functions in a *black-box manner* is $\Omega (1/\sqrt{n})$-unfair, settling this long-standing open problem and proving the qualitative optimality of the protocol by [34,35,36,37]. Black-box separation is a prominent technique introduced by Impagliazzo and Rudich [31], and further nuances in its definition were highlighted in [32,70]. Suppose one “*black-box separates* the cryptographic primitive *Q* from another cryptographic primitive *P*.” Then, one interprets this result as indicating that the primitive *P* is unlikely to facilitate the secure construction of *Q* using black-box constructions. Most constructions in theoretical computer science and cryptography are black-box in nature. That is, they rely only on the input–output behavior of the primitive *P*, and are oblivious to, for instance, the particular implementation of the primitive *P*. The security reduction in cryptographic black-box constructions also uses the adversary in a black-box manner. There are, however, some highly non-trivial nonblack-box constructions in theoretical computer science, for example, [57,71,72,73,74,75,76,77]. However, an infeasibility of black-box constructions to realize *Q* from *P* indicates the necessity of new nonblack-box constructions, which, historically, have been significantly infrequent. Prior to this result, this black-box separation was known only for restricted families of constructions [11,12,78,79].

Subsequently, using similar techniques, ref. [30] further strengthens this separation beyond Minicrypt by proving that any fair coin-tossing protocol using a public-key encryption algorithm (which is even stronger than one-way functions) in a black-box manner is also $\Omega (1/\sqrt{n})$-unfair. In fact, ref. [30] proves a stronger result. Let *f* be a secure function evaluation functionality, such that (two-choose-one single-bit) oblivious transfer [63,64] can be securely implemented in the *f*-hybrid model (i.e., a system where parties have access to an idealized implementation of the *f*-functionality). In this *f*-hybrid model, one can emulate the optimal fair coin-tossing protocol of Moran, Naor, and Segev [67], which is O(1/n)-unfair. However, consider *f*, such that oblivious transfer is impossible in the *f*-hybrid model (all such functions were characterized by [80]). In the *f*-hybrid model, ref. [30] proves that any fair coin-tossing protocol using a public-key encryption protocol in a black-box manner is $\Omega (1/\sqrt{n})$-unfair. These results prove that the set of all secure function evaluation functionalities have the following dichotomy. Given any secure function evaluation *f*, either there exists an optimal fair coin-tossing protocol in the *f*-hybrid model, or any coin-tossing protocol (even using public-key encryption in a black-box manner) in the *f*-hybrid model is $\Omega (1/\sqrt{n})$-unfair.

### 4.2. Augmented Protocols

Discussion

Let Π be a coin-tossing protocol in the information-theoretic plain model. Assume that *T* is the partial transcript of the protocol. Suppose $V_{A}$ is the random variable denoting the private view of Alice, and $V_{B}$ is the random variable denoting the private view of Bob. Then, the following Markov chain is an essential property of protocols in the information-theoretic plain model.

$$V_{A}↔T↔V_{B}.$$

That is, conditioned on the partial transcript *T*, the joint distribution of Alice–Bob private views $\left(V_{A},V_{B}|T\right)$ is a product of the marginal distributions of their views, that is $\left(V_{A}|T\right)\times \left(V_{B}|T\right)$. However, when parties have access to an oracle, then this property need not hold.

Augmented Protocols

In the random oracle model, it is not necessary that the joint distribution of Alice–Bob private views $\left(V_{A},V_{B}|T\right)$ is a product of the marginal distributions of their views. Their views may be correlated via the random oracle and this correlation is not eliminated by public transcript. For example, if the private queries to the random oracle by Alice and Bob are disjointed, then the joint distribution of their private views is a product distribution. However, if they have common private queries then, conditioned on the public transcript, their views may be correlated. For example, suppose Alice and Bob privately query at 0. In this case, conditioned on the public transcript, the answer to the query has *n*-bits of entropy. The views of Alice and Bob are perfectly correlated, i.e., the mutual information of their private views is *n*.

There are standard techniques to circumvent this bottleneck. There exists a *public querying algorithm* (one that depends only on the public transcript of the protocol) and performs additional queries to the random oracle and can reduce this correlation. In particular, for any ε∈(0,1), there are public algorithms [31,62,81] that perform poly(n/ε) additional queries to the random oracle, such that, (with high probability) conditioned on these additional query–answer pairs, the joint distribution of Alice–Bob views is ε-close (in the statistical distance) to the product of its marginal distributions. We emphasize that the parameter ε may depend on the input-length *n* of the random oracle and the round complexity *r* of the protocol.

Consequently, any protocol in the random oracle model can be converted into an augmented protocol where the party sending the next message in the protocol adds the query–answer pairs of the public querying algorithm mentioned above. This compilation preserves the round complexity of the protocol while ensuring that the crucial Markov chain property holds. Therefore, for the rest of this section, we shall assume that the coin-tossing protocol in the random oracle model is an augmented protocol.

### 4.3. Technical Proof

Recall that, in augmented coin-tossing protocols, the joint distribution of Alice–Bob views is ε close to the product of their marginal distribution with high probability. For the simplicity of presentation, we shall assume that $V_{A}↔T↔V_{B}$ for augmented coin-tossing protocols in the random oracle model. The simplified analysis captures all the primary innovations of the new proof strategy of Maji and Wang [16,30]. The actual analysis only introduces a poly(ε) slack in the simplified analysis presented below.

Main Result and Inductive Hypothesis

Our objective is to prove the following result.

**Theorem** **2.** *There exists a universal constant C>0, such that any two-party r-message bias-X fair coin-tossing protocol in the random oracle model is at least $\frac{C}{\sqrt{r}}\cdot X\left(1-X\right)$-unfair.*

This result, proved in [16], implies that any coin-tossing protocol using one-way functions in a (fully) black-box manner is $\Omega (1/\sqrt{r})$-unfair. [30] extends this result to demonstrate a separation from public-key encryption even in an *f*-hybrid model, where *f* is a secure function evaluation functionality, such that secure oblivious transfer does not exist in the *f*-hybrid model.

Towards proving this theorem, the survey shall summarize the proof of the following technical result. Consider a stopping time τ:Ω→{1,2,…,n,∞}. Let $X_{\tau }$ represent the expected color conditioned on the partial transcript $\left(T_{1},\;\ldots ,\;T_{\tau }\right)$. Similarly, $A_{\tau }$ represents the expected Alice defense conditioned on the fact that Bob aborts at the partial transcript $\left(T_{1},\;\ldots ,\;T_{\tau }\right)$. Analogously, $B_{\tau }$ represents the expectation of the Bob defense coin conditioned on the fact that Alice aborts at the partial transcript $\left(T_{1},\;\ldots ,\;T_{\tau }\right)$.

**Theorem** **3.** *There exists a universal constant C>0, such that for any two-party r-message bias-X, the fair coin-tossing protocol in the random oracle model the following bound holds.*

$$\underset{\mathrm{stopping}\;\mathrm{time}\;\tau }{\sup}E[|X_{\tau }-A_{\tau }\left|+\right|X_{\tau }-B_{\tau }|]\geq \frac{4C}{\sqrt{n}}\cdot X\left(1-X\right).$$

Given Theorem 3 it is easy to prove Theorem 2 using an averaging argument. The stopping time τ, witnessing the attack inTheorem 3, accounts for four different attacks: Alice/Bob increasing/decreasing the expected output of the coin-tossing protocol. So, one of these four attacks changes the expected output of the coin-tossing protocol by at least $\frac{C}{\sqrt{n}}\cdot X\left(1-X\right).$

Potential Function

Our analysis shall use the following potential function.

$$\Phi \left(x,a,b\right):=x\left(1-x\right)+{\left(x-a\right)}^{2}+{\left(x-b\right)}^{2}.$$

Looking ahead, the variable *x* shall represent the expected output conditioned on the partial transcript being *T*. Furthermore, *a* shall represent the expected Alice defense coin (i.e., Alice output if Bob aborts) conditioned on the partial transcript being *T*. Similarly, *b* shall represent the expected Bob defense coin conditioned on the partial transcript being *T*. Intuitively, in hindsight, the term x(1−x) in the potential represents the quality of the fail-stop attack, owing to the entropy in the output. For example, if the expected output *x* is already close to 0 or 1, it is possible that one cannot significantly change the output of the protocol. However, if the expected output *x* is far from both 0 and 1, then we shall show that a large attack is possible. Furthermore, the term ${\left(x-a\right)}^{2}$, intuitively, captures the quality of the attack on honest Alice if she defends improperly. For example, if Bob aborts, then the output distribution of Alice changes by |x−a|. Similarly, the term ${\left(x-b\right)}^{2}$ captures the quality of the attack on honest Bob if his expected defense is far from the expected output.

We remark that the function Φ(·,·,·) is *not* convex, in general. However, Jensen’s inequality holds when one considers the evolution of augmented coin-tossing protocols.

Convexity of the Potential Function in Augmented Protocols

Given a partial transcript *T* of an augmented protocol, conditioned on this partial transcript *T*, let (1) *X* represent the expected output of the protocol; (2) *A* be the expected Alice defense coin; (3) *B* be the expected Bob defense coin. Consider one step in the evolution of the protocol. Let $T^{\prime }$ be the random variable representing the transcript that extends the transcript *T* by one step in the protocol evolution. We represent this randomized evolution as $T^{\prime }⊢T$. Let $X^{\prime },A^{\prime },B^{\prime }$ represent the expected output, Alice defense, and Bob defense, respectively, conditioned on the partial transcript $T^{\prime }$.

Observe that the following identities, referred to as the *martingale properties*, hold.

$$\begin{array}{cc} E_{T^{\prime }⊢T}\left[X^{\prime }\right] & =X\\ E_{T^{\prime }⊢T}\left[A^{\prime }\right] & =A,\;\mathrm{and}\\ E_{T^{\prime }⊢T}\left[B^{\prime }\right] & =B. \end{array}$$

The augmented protocol has the property that the joint distribution of the views of Alice and Bob is *close* to the product of its marginal distributions. In the sequel, for the simplicity of presentation, we shall assume that the joint distribution of the Alice and Bob views is *identical* to the product of its marginal distributions. This simplifying assumption essentially preserves the qualitative nature of our results. The actual analysis incurs a small slack in the lower bound that is linear in the “closeness parameter.” Using our simplifying assumption, we have the following identity in augmented fair coin-tossing protocols.

$$E_{T^{\prime }⊢T}\left[A^{\prime }\cdot B^{\prime }\right]=E_{T^{\prime }⊢T}\left[A^{\prime }\right]\cdot E_{T^{\prime }⊢T}\left[B^{\prime }\right].$$

Now, we can state and prove a Jensen’s inequality for our potential function that holds only in augmented fair coin-tossing protocols.

**Proposition** **1** (Jensen’s Inequality for Augmented Protocols). *The following inequality holds in any augmented fair coin-tossing protocol.*

$$E_{T^{\prime }⊢T}\left[\Phi \left(X^{\prime },A^{\prime },B^{\prime }\right)\right]\geq \Phi \left(X,A,B\right).$$

**Proof.**  Consider the following manipulation.

$$\begin{array}{ccc} E_{T^{\prime }⊢T}\left[\Phi \left(X^{\prime },A^{\prime },B^{\prime }\right)\right] & =E_{T^{\prime }⊢T}\left[X^{\prime }\left(1-X^{\prime }\right)+{\left(X^{\prime }-A^{\prime }\right)}^{2}+{\left(X^{\prime }-B^{\prime }\right)}^{2}\right] & \left(\mathrm{expanding}\right)\\ \; & =E_{T^{\prime }⊢T}\left[X^{\prime }+{\left(X^{\prime }-A^{\prime }-B^{\prime }\right)}^{2}-2A^{\prime }B^{\prime }\right] & \left(\mathrm{rearranging}\right)\\ \; & =E_{T^{\prime }⊢T}\left[X^{\prime }\right]+E_{T^{\prime }⊢T}\left[{\left(X^{\prime }-A^{\prime }-B^{\prime }\right)}^{2}\right]-2E_{T^{\prime }⊢T}\left[A^{\prime }B^{\prime }\right] & \left(\mathrm{linearity}\;\mathrm{of}\;\mathrm{expectation}\right)\\ \; & =X+E_{T^{\prime }⊢T}\left[{\left(X^{\prime }-A^{\prime }-B^{\prime }\right)}^{2}\right]-2E_{T^{\prime }⊢T}\left[A^{\prime }B^{\prime }\right] & \left(\mathrm{martingale}\;\mathrm{property}\right)\\ \; & \geq X+E_{T^{\prime }⊢T}{\left[\left(X^{\prime }-A^{\prime }-B^{\prime }\right)\right]}^{2}-2E_{T^{\prime }⊢T}\left[A^{\prime }B^{\prime }\right] & \left({\mathrm{Jensen}}^{\prime }s\;\mathrm{inequality}\;\mathrm{on}\;\mathrm{the}\;\mathrm{function}\;Z^{2}\right)\\ \; & =X+{\left(X-A-B\right)}^{2}-2E_{T^{\prime }⊢T}\left[A^{\prime }B^{\prime }\right] & \left(\mathrm{martingale}\;\mathrm{property}\right)\\ \; & =X+{\left(X-A-B\right)}^{2}-2E_{T^{\prime }⊢T}\left[A^{\prime }\right]\cdot E_{T^{\prime }⊢T}\left[B^{\prime }\right] & \left(\mathrm{augmented}\;\mathrm{protocol}\;\mathrm{property}\right)\\ \; & =X+{\left(X-A-B\right)}^{2}-2AB & \left(\mathrm{martingale}\;\mathrm{property}\right)\\ \; & =\Phi (X,A,B). & \left(\mathrm{rearranging}\right) \end{array}$$

This observation completes the proof of the claim. □

A Technical Lemma

For our proof, we shall need a technical lemma.

**Lemma** **1** (Technical Lemma: Quality of Choice). *There exists a universal positive constant C, such that the following identity holds.*

$$\max\left\{|X^{\prime }-A^{\prime }\left|+\right|X^{\prime }-B^{\prime }|,\frac{4C}{\sqrt{n-1}}\cdot X^{\prime }\left(1-X^{\prime }\right)\right\}\geq \frac{4C}{\sqrt{n}}\cdot \Phi \left(X^{\prime },A^{\prime },B^{\prime }\right).$$

The interested reader may refer to [15] for a proof of this technical result. For $A^{\prime }=B^{\prime }=X$, this result is an algebraization of the geometric transformation in Figure 6 while introducing some slack in the constants.

Putting things together: The Inductive Argument

**ProofofTheorem3**  We present the proof of the inductive step of the result. Let T=∅, the empty transcript, and $T^{\prime }$ be the first message of the augmented coin-tossing protocol. Below, we use $\Pi^{\prime }$ to represent the coin-tossing protocol Π conditioned on the partial transcript, which is $T^{\prime }$.

$$\begin{array}{ccc} \mathrm{opt}(\Pi ) & =E_{\Pi^{\prime }⊢\Pi }\left[\max\left\{|X^{\prime }-A^{\prime }]\left|+\right|X^{\prime }-B^{\prime }|,\mathrm{opt}\left(\Pi^{\prime }\right)\right\}\right] & \left(\mathrm{definition}\;\mathrm{of}\;\mathrm{the}\;\mathrm{optimal}\;\mathrm{adversarial}\;\mathrm{strategy}\right)\\ \; & \geq E_{\Pi^{\prime }⊢\Pi }\left[\max\left\{|X^{\prime }-A^{\prime }]\left|+\right|X^{\prime }-B^{\prime }|,\frac{4C}{\sqrt{n-1}}\cdot X^{\prime }\left(1-X^{\prime }\right)\right\}\right] & \left(\mathrm{inductive}\;\mathrm{hypothesis}\right)\\ \; & \geq E_{\Pi^{\prime }⊢\Pi }\left[\frac{4C}{\sqrt{n}}\cdot \Phi \left(X^{\prime },A^{\prime },B^{\prime }\right)\right] & \left(\mathrm{technical}\;\mathrm{lemma}\right)\\ \; & \geq \frac{4C}{\sqrt{n}}\cdot \Phi \left(X,A,B\right) & \left({\mathrm{Jensen}}^{\prime }s\;\mathrm{inequality}\;\mathrm{for}\;\mathrm{our}\;\mathrm{potential}\;\mathrm{function}\right)\\ \; & =\frac{4C}{\sqrt{n}}\cdot \left(X\left(1-X\right)+{\left(X-A\right)}^{2}+{\left(X-B\right)}^{2}\right) & \left(\mathrm{definition}\;\mathrm{of}\;\mathrm{the}\;\mathrm{potential}\;\mathrm{function}\right)\\ \; & \geq \frac{4C}{\sqrt{n}}\cdot X\left(1-X\right) & \left(\mathrm{non}-\mathrm{negativity}\;\mathrm{of}{\left(X-A\right)}^{2}\;\mathrm{and}{\left(X-B\right)}^{2}\right) \end{array}$$

This derivation concludes the proof of our main technical result. □

## 5. Isoperimetric Inequalities

This section considers *n*-processors one-turn one-round coin-tossing protocols. The strong [33] Byzantine adversary can see all messages of the processors and then decide to corrupt *k* processors of its choice. Let $\varepsilon^{+}\left(\pi \right)$ represent the maximum insecurity caused by a Byzantine adversary who increases the expected output of the protocol. Similarly, $\varepsilon^{-}\left(\pi \right)$ represents the maximum insecurity caused by a Byzantine adversary decreasing the expected output.

The connection to isoperimetric inequalities [3,4,5,82] (via the expansion of fixed density subset of product spaces) establishes the relevance to topics in theoretical computer science, such as expander graphs, complexity theory, and error-correcting codes. Every coin-tossing protocol is equivalent to a unique subset *S* of an *n*-dimension product space $\Sigma^{n}$, where the size of the alphabet set σ:=|Σ| depends on the randomness complexity of the coin-tossing protocol. In the full information model, without the loss of generality, one can assume that all interactive protocols are stateless, and processors use a fresh block of private randomness to generate the next message at any point during the evolution of the coin-tossing protocol [83,84,85]. Furthermore, each processor can directly broadcast its local private randomness as her message because the final output is a deterministic function of the public transcript. Elements of this product space represent the complete transcript of the coin-tossing protocol, the *i*-th coordinate of an element corresponds to the message sent by processor *i*, and the subset *S* contains all elements of the product space on which the coin-tossing protocol outputs 1. One considers the uniform distribution over $\Sigma^{n}$ to sample the elements.

The discussion in the sequel extends to the arbitrary corruption threshold *k*. However, for the simplicity of the presentation, we consider the specific case of k=1. Let $\partial S_{k}^{+}$ be the set of elements in $\bar{S}$ (the complement of *S*) that are at a Hamming distance k=1 from the set *S*. Consequently, a Byzantine adversary can change an element from the set $\partial S_{k}^{+}\subseteq \bar{S}$ into some element of *S* by editing (at most) k=1 coordinates. Note that, if the Byzantine adversary can see *all* the messages and then performs the edits, then it can increase the expected output by exactly $\varepsilon^{+}=\left|\partial S_{k}^{+}\right|/\sigma^{n}$.

Analogously, one defines the set $\partial S_{k}^{-}\subseteq S$ that contains all elements at a Hamming distance k=1 from the set $\bar{S}$. So, a Byzantine adversary who sees all the messages before editing can reduce the expected output by $\varepsilon^{-}=\left|\partial S_{k}^{-}\right|/\sigma^{n}$.

Traditional isoperimetric inequalities over product spaces consider either the edge or vertex perimeter of the set *S*. The vertex perimeter of a set is most relevant to our current discussion. In this extremal graph-theoretic terminology, the (width-*k*) *vertex perimeter* of the set *S*, represented by $\partial_{V,k}S$ is the set of all elements in $\bar{S}$ that are at a Hamming distance of at most *k* from some element in *S*. Therefore, the perimeter $\partial_{V,k}S$ is identical to the set $\partial S_{k}^{+}$. Similarly, the vertex perimeter of the set $\bar{S}$ (which is $\partial_{V,k}\bar{S}$) is identical to the set $\partial S_{k}^{-}$.

Extremal graph theory studies the vertex perimeter of a dense set *S*; the density of the set *S* is *X* if this set corresponds to a bias-*X* coin-tossing protocol. The density of the set $\bar{S}$, therefore, is (1−X). The objective of extremal graph theory is to characterize the optimal set *S* of a fixed density that minimizes its vertex perimeter. That is, equivalently, the objective is to design a coin-tossing protocol (identified by *S*) that minimizes $\varepsilon^{+}$. Note that minimizing $\varepsilon^{+}$ does *not* automatically entail the simultaneous minimization of $\varepsilon^{-}$ for general Σ.

For small alphabet-size, for example, when σ=2, the choice of *S* that minimizes its perimeter is explicitly known (namely, the set of appropriate density that greedily includes the smallest elements in the simplistic ordering). For this value of σ, *it happens to be the case* that the complementary set of *S* simultaneously minimizes $\varepsilon^{-}$. For large alphabets, however, there are explicit counterexamples. Consider the large alphabet set Σ={0,1,…,σ−1}, for a suitable choice of σ. Each party broadcast a random element from the set. The AND protocol checks if all messages are non-zero. This protocol realizes $\left(\varepsilon^{+},\varepsilon^{-}\right)=\left(\frac{1-X}{n},X\right)$. The complementary protocol realizes $\left(\varepsilon^{+},\varepsilon^{-}\right)=\left(1-X,\frac{X}{n}\right)$. Therefore, there are coin-tossing protocols achieving $\left(\varepsilon^{+},\varepsilon^{-}\right)=\left(\frac{1-X}{n},X\right)$ and $\left(\varepsilon^{+},\varepsilon^{-}\right)=\left(1-X,\frac{X}{n}\right)$), demonstrating that minimizing the perimeter of *S* does not automatically minimize the perimeter of $\bar{S}$.

The *new cryptographic objective* is to minimize $\varepsilon =\max\{\varepsilon^{+},\varepsilon^{-}\}$, i.e., simultaneously minimize the maximum of the vertex perimeters of *S* and $\bar{S}$. One may choose the *proxy objective* of minimizing the sum of the perimeters of *S* and $\bar{S}$, that is, the quantity $\varepsilon^{+}+\varepsilon^{-}$. This proxy objective is a two-approximation of the new cryptographic objective.

In particular, this observation motivates studying new isoperimetric inequalities in extremal graph theory that are inspired by natural practical applications. Instead of minimizing the vertex perimeter of a set *S* of fixed density, one considers the new objective of minimizing the *symmetric perimeter* defined under various norms.

$$\partial_{V,k,\ell }^{\mathrm{sym}}\left(S\right){\left(|\partial_{V,k}S{|}^{\ell }+{\left|\partial_{V,k}\bar{S}\right|}^{\ell }\right)}^{1/\ell }.$$

The ℓ=∞ case corresponds to our new cryptographic objective, and the ℓ=1 corresponds to the case above. The proposed research provides evidence that such symmetric perimeters may be more well-behaved in general. For instance, when k=1 and ℓ=1, Khorasgani, Maji, and Wang [29] demonstrate that the density of the symmetric perimeter is $1/\sqrt{n}$ for any dense set *S*, even in product spaces over large alphabets.

## 6. Conclusions

There are several fascinating open problems in coin-tossing protocols pertaining to the topics covered in this survey. This section highlights two such problems below.

Consider a coin-tossing protocol where processors have multiple turns. In a *t*-turn coin-tossing protocol, every processor sends messages in *t* rounds. Consequently, once the adversary corrupts a particular processor, it can adversarially set all its future messages. In this setting, what is the optimal bias-*X* coin-tossing protocol? For two processors, we know that one of the processors can force an output 0/1 with certainty (based on games against nature [68]). For a larger number of processors, characterizing the optimal coin-tossing protocols remains open.

For the relativized separation results, [30] proved that if oblivious transfer is impossible in the *f*-hybrid model, then any *r*-round coin-tossing protocol in the *f*-hybrid model is (roughly) $\frac{C}{\sqrt{r}}$-unfair. Fix one such function *f* that does not allow for the oblivious transfer for the discussion below. Now, consider the hardness of computation assumption that “there exists a secure protocol for *f*.” Observe that this hardness of computation assumption is as powerful as the *f*-hybrid model. However, it may implicitly provide additional restrictions on the computation power of the parties and the adversaries. Consider the following analogy from complexity theory to highlight this subtle difference. In the first scenario, consider efficient algorithms with access to the NP-oracle. In the second scenario, consider the assumption that NP = P. The second scenario is at least as powerful as the first scenario. However, the second scenario has several additional implicit consequences. In particular, the entire PH collapses, and we have P=PH. Consequently, is it possible that, through the hardness of computation assumption “there exists a secure protocol for *f*,” which is stronger than the *f*-hybrid model and enables coin-tossing with lower unfairness?

## Figures and Tables

**Table 1:** State-of-the-art constructions and hardness of computation results in fair coin-tossing. The expression “f↛OT” represents the fact that there is no secure protocol for oblivious transfer [63,64] in the *f*-hybrid model against honest-but-curious adversaries.

	Secure Construction	Adversarial Attack
Pessiland		In General: Ω(1)-unfair [65,66]
		Fail-stop Adversary: $\Omega (1/\sqrt{n})$-unfair [10,14]
Minicrypt	One-way Functions: $O(1/\sqrt{n})$-unfair [34,35,36,37]	One-way Functions: $\Omega (1/\sqrt{n})$-unfair [16]
Cryptomania	Public-key Encryption:	Public-key Encryption: $\Omega (1/\sqrt{n})$-unfair [30]
	PKE + *f*-hybrid, f↛OT:	PKE + *f*-hybrid, f↛OT: $\Omega (1/\sqrt{n})$-unfair [30]
	Oblivious Transfer: O(1/n)-unfair [67]	Oblivious Transfer: Ω(1/n)-unfair [37]

## Data Availability

Data sharing not applicable.
